# Accuracy of Selection in Early Generations of Field Pea Breeding Increases by Exploiting the Information Contained in Correlated Traits

**DOI:** 10.3390/plants12051141

**Published:** 2023-03-02

**Authors:** Felipe A. Castro-Urrea, Maria P. Urricariet, Katia T. Stefanova, Li Li, Wesley M. Moss, Andrew L. Guzzomi, Olaf Sass, Kadambot H. M. Siddique, Wallace A. Cowling

**Affiliations:** 1 The UWA Institute of Agriculture, The University of Western Australia, Perth, WA 6009, Australia; 2 School of Agriculture and Environment, The University of Western Australia, Perth, WA 6009, Australia; 3 General Genetics Unit, Pontificia Universidad Católica Argentina, Buenos Aires C1107AAZ, Argentina; 4 SAGI West, School of Molecular and Life Sciences, Curtin University, Perth, WA 6845, Australia; 5 Animal Genetics and Breeding Unit, University of New England, Armidale, NSW 2351, Australia; 6 Centre for Engineering Innovation: Agriculture & Ecological Restoration, The University of Western Australia, Shenton Park, WA 6008, Australia; 7 School of Engineering, The University of Western Australia, Perth, WA 6009, Australia; 8 Norddeutsche Pflanzenzucht Hans-Georg Lembke KG, Hohenlieth-Hof 1, 24363 Holtsee, Germany

**Keywords:** stem strength, ascochyta blight disease complex, black spot, *Didymella pinodes*, ABLUP, multivariate, multi-trait, linear mixed models, optimal contribution selection, estimated breeding value, accuracy, non-inbred progeny, EBV, PBV

## Abstract

Accuracy of predicted breeding values (PBV) for low heritability traits may be increased in early generations by exploiting the information available in correlated traits. We compared the accuracy of PBV for 10 correlated traits with low to medium narrow-sense heritability ($h^{2})$ in a genetically diverse field pea (*Pisum sativum* L.) population after univariate or multivariate linear mixed model (MLMM) analysis with pedigree information. In the contra-season, we crossed and selfed S_1_ parent plants, and in the main season we evaluated spaced plants of S_0_ cross progeny and S_2+_ (S_2_ or higher) self progeny of parent plants for the 10 traits. Stem strength traits included stem buckling (SB) ($h^{2}$ = 0.05), compressed stem thickness (CST) ($h^{2}$ = 0.12), internode length (IL) ($h^{2}$ = 0.61) and angle of the main stem above horizontal at first flower (EAngle) ($h^{2}$ = 0.46). Significant genetic correlations of the additive effects occurred between SB and CST (0.61), IL and EAngle (−0.90) and IL and CST (−0.36). The average accuracy of PBVs in S_0_ progeny increased from 0.799 to 0.841 and in S_2+_ progeny increased from 0.835 to 0.875 in univariate vs MLMM, respectively. An optimized mating design was constructed with optimal contribution selection based on an index of PBV for the 10 traits, and predicted genetic gain in the next cycle ranged from 1.4% (SB), 5.0% (CST), 10.5% (EAngle) and −10.5% (IL), with low achieved parental coancestry of 0.12. MLMM improved the potential genetic gain in annual cycles of early generation selection in field pea by increasing the accuracy of PBV.

## 1. Introduction

It is important to increase the rate of genetic gain in self-pollinating crops to meet future demand for grains, and the most promising way to increase the rate of gain is to shorten selection cycles [1]. This may be achieved through selection on early generation non-inbred progeny [1]. However, selection on non-inbred progeny is less accurate than on inbred lines [2,3,4], and therefore crop breeders normally prefer to self to purity before choosing parents for crossing. This extends selection cycles and reduces the potential rate of genetic gain. In this study, we explore methods which increase the accuracy of predicted breeding values (PBVs) for low to medium heritability traits in non-inbred progeny. The goal was to shorten selection cycles and potentially accelerate genetic gain.

One of the most important grain legume crops worldwide is field pea (*Pisum sativum* L.), with production of over 14 million tonnes on more than seven million hectares globally [5]. Peas are highly adaptable to a wide range of soils and are used for both animal feed and human consumption. The fiber, vitamin, and protein content in pea grains makes them particularly desirable for human diets and it is likely that this crop will play a key role in securing future food production and human nutrition [6,7].

One of the main limiting factors in field pea production is its susceptibility to ascochyta blight disease complex, reported to reduce yields by as much as 70% in some regions [8,9,10,11]. Breeding for resistance to this complex trait has not been highly successful, due to the different fungal pathogens involved [12,13], the low level of resistance available, and polygenic inheritance of resistance [8]. So far, released varieties do not show an adequate level of resistance [8,10,14,15]. Moderate levels of resistance were observed in some wild relatives and breeding populations of field pea [14,16,17]. Low to moderate narrow-sense heritability was reported for ascochyta blight resistance in an experimental pea population based on diverse sources of partial resistance [9]. Breeding schemes based on pedigree information [18] and, if available, genomic tools [13,19] will increase the potential for future genetic gain in ascochyta blight resistance.

Another constraint in pea production is canopy lodging, which not only promotes disease spread but also reduces harvestability, leading to yield loss and higher costs for farmers [20,21]. Improvements in lodging resistance have been mostly achieved by modifying pea architecture with dwarf semi-leafless varieties [7,15,22] which improve the standability of the crop thanks to tendrils holding the canopy together [23]. However this trait depends on plant-to-plant interaction in the crop and may not reflect stem strength of individual pea plants. Lodging resistance has been associated also with thicker stem walls and higher kinetic energy required to cut stems [24]. Stem strength in field pea, measured as flexion and force at breaking point, was correlated with compressed stem thickness and stem diameter, but heritability for these traits was low [20,25].

The rate of genetic gain for major crops is around 1% per annum and is insufficient to meet global food demands by 2050 [26] and must be at least doubled to meet the increasing global demand for food production [27,28,29]. Cobb et al. [1] reviewed the effect of changes in components of the breeder’s equation and concluded that the most likely way to improve the rate of genetic gain in crop breeding programs is to reduce cycle length. This can be done by shortening selfing generations to rapidly generate inbred lines [30,31], or by selecting non-inbred progeny as parents to begin the next cycle [1]. It should be possible to improve accuracy of PBVs for low heritability traits in non-inbred progeny by exploiting information gained from correlated traits and pedigree and/or genomic information [2,3,32,33].

Multivariate maximum likelihood models were developed for animal breeding in the 1980s, and were shown to improve the data structure and increase the accuracy of PBV for correlated traits compared to univariate models [34]. A single-step multivariate linear mixed model (MLMM) with pedigree information was developed in a cross-pollinating tree species and combined structured variance-covariance matrices at the treatment and residual levels to improve accuracy of PBV [35]. Multivariate analysis in crops has so far been applied to improve model prediction accuracy in inbred wheat and barley lines [36,37,38,39] and doubled haploid maize lines [40], but this is the first study to evaluate accuracy of PBV with MLMM in non-inbred progeny of a self-pollinating crop.

The exploitation of correlated traits is useful when one of the traits is difficult or expensive to measure [41] or has lower heritability [2,42]. Additionally, the measurements may be components of one trait, such as disease severity measured by two correlated traits, crop density before and after disease development, and bivariate analysis was shown to improve the accuracy of genetic values for both traits [43]. The benefits of MLMM over univariate analysis will depend on the heritabilities and correlations between traits and environments [2]. A strong correlation between traits will improve model prediction, even if the correlation is unfavourable [39]. However, a multivariate model based on many traits with low or no correlation may have no benefits over univariate models [2,38].

Significant gains were made in ascochyta blight resistance across two cycles of recurrent selection based on non-inbred progeny of field pea in an animal model analysis across cycles [18]. Here, we re-analyze the data from Cowling et al. [18] to select parents to begin the next cycle of this experimental pea population, and we apply MLMM to select non-inbred progeny for several low to moderate heritability traits. We combine random and/or fixed spatial effects and covariances of additive, non-additive and residual effects in the analysis of ascochyta blight resistance, flowering time, stem strength traits, grain yield, biomass, and other important agronomic traits.

The aim of this paper is to improve accuracy of PBV in non-inbred progeny of field pea by exploiting the information available in correlated low to moderate heritability traits. This is one of the first studies to use MLMM in crop breeding for this purpose. We hypothesize that accuracy of PBVs for these correlated low to medium heritability traits will be higher in MLMM than in a univariate linear mixed model (LMM), and compare the accuracy of PBVs in S_0_ progeny with self progeny of parent plants (S_2_ or higher selfing level) evaluated in the same experiment. In a novel approach to test the value of the PBV generated by MLMM, we predict the genetic gain of traits and achieved parental coancestry in the next cycle with optimal contribution selection (OCS) [44] based on an index of MLMM-derived PBVs weighted to achieve desired genetic gains.

## 2. Results

For definition of abbreviations, please see Section 4 Materials and Methods.

### 2.1. Re-Analysis of 2015 Data (Cycle 2) and Mating Design for Cycle 3

The bivariate LMM analysis of ABS and DTF in Cycle 2 2015 revealed a negative genetic correlation of additive effects (r = −0. 64) between ABS and DTF (Supplemental Table S1A,B). Narrow-sense heritability ($h^{2}$) for ABS was 0.377 and average accuracy ± standard deviation of PBV for ABS in S_0_ progeny was 0.826 ± 0.04, which is slightly higher than 0.805 reported from univariate analysis in Cowling et al. [18]. $h^{2}$ for DTF was 0.555 and average accuracy in S_0_ progeny was 0.850 ± 0.04. The PBVs obtained from this model were given appropriate economic weights in a selection index, and the index was used to generate an optimised mating design for Cycle 3 based on OCS (Supplemental Table S1C,D).

### 2.2. 2019(Cycle 3) Trial Data Preparation

**Figure 1:**
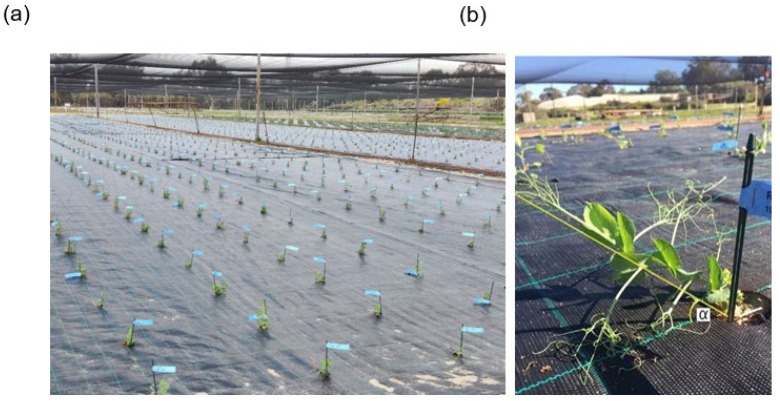
Field trial of Cycle 3 experimental field pea population in UWA field station, Shenton Park 6008, Western Australia in June 2019. (**a**) 1600 single plant plots sown in a square grid design partially replicated trial, comprising 668 S_0_ genotypes, 612 S_2+_ genotypes and 320 control plants (replicates of 15 control varieties); (**b**) close-up of a single plant immediately before first flower showing the stem angle above horizontal (EAngle) measured in degrees above horizontal (α).

**Figure 2:**
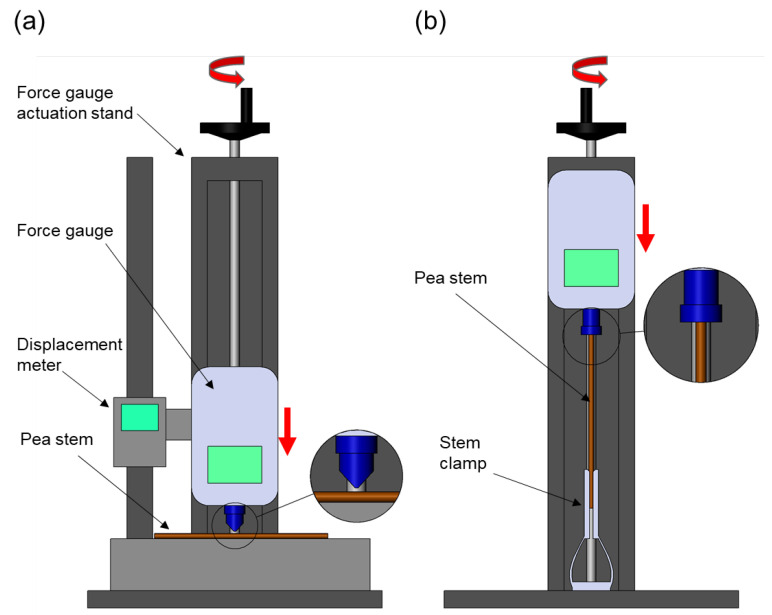
Diagram of measurement conditions for (**a**) compressed stem thickness (CST) and (**b**) stem buckling (SB) in dry field pea stems. In both settings the force gauge was actuated downwards, applying a load over the pea stem. In (**a**) a displacement meter was used to measure the displacement of the stem from its natural stem diameter (SD) to the CST after applying a load of 10 N. For (**b**) the bottom of the stem was restricted with a clamp and the top was fixed under the flat end of the force gauge. SB was measured as the peak force recorded at stem failure.

Data for 10 traits and 1600 entries were assessed in Cycle 3 2019 (Figure 1a, Supplemental Table S2A,B). EAngle was measured before flowering, when stem bending began to occur in plots (Figure 1b). DTF, ABS and Br were measured throughout the season. GY and BM were measured after harvest on dry plants. SD, IL, CST (Figure 2a), and SB (Figure 2b) were measured on dry main stems after harvest as described in Section 4 Materials and Methods.

### 2.3. Univariate Linear Mixed Model Analyses of 2019 Data (Cycle 3)

Single traits were analysed first in univariate LMM without pedigree information, providing estimates of the total genotype and residual variance for each trait, and then with pedigree information to estimate additive (and potentially non-additive) variance components and narrow-sense heritability for each trait (Table 1, Supplemental Table S3). In all univariate models, linear fixed effects for range and row were not significant. $h^{2}$ for GY, BM, SB, SD, CST, and ABS was low ($h^{2}$ ≤ 0.15), low to moderate for Br and EAngle ($h^{2}$ ≤ 0.36) and moderate for DTF and IL ($h^{2}$ > 0.50) (Table 1).

### 2.4. Multivariate Linear Mixed Model Analyses

#### 2.4.1. 10-Trait Model

The analysis of all 10 traits in the MLMM in ASReml-R achieved convergence with a US variance structure for additive genetic effects and residual effects, while omitting any other form of random effect. However, US variance structures were modified in all iterations of the run to maintain positive definite values (Supplemental Table S4A). Hence, the optimised MLMM was completed in the stand-alone version of ASReml by first adding the significant components for each trait identified in the univariate LMM and later removing those components that became non-significant under the new variance structure in the MLMM. In the optimized MLMM, variance components for additive genetic effects increased for GY, BM, Br, DTF, EAngle, and IL (Table 2) compared to the estimates of the univariate model (Table 1). ABS had a slightly smaller variance component for the additive genetic effect. The variance components of residual effects increased for all traits in the optimized MLMM, except for EAngle (Table 2). In the optimized MLMM, non-additive genetic effects were significant only for GY and DTF, and these effects were assumed independent (DIAG variance structure was used for non-additive effects). Compared to the univariate models (Table 1), $h^{2}$ in the MLMM (Table 2) improved considerably for DTF (increased by 0.10 compared to univariate LMM), EAngle (increased by 0.23) and IL (increased by 0.08) and increased slightly for GY (increased by 0.01). In contrast, $h^{2}$ decreased slightly for the other traits (−0.01 to −0.02) (Table 1 and Table 2). The standard errors of $h^{2}$ were similar for all traits in univariate LMM and MLMM except for EAngle, where the standard error of $h^{2}$ increased from 0.04 to 0.12 (full model components can be seen in Supplemental Table S4B).

#### 2.4.2. Trait Correlations

**Figure 3:**
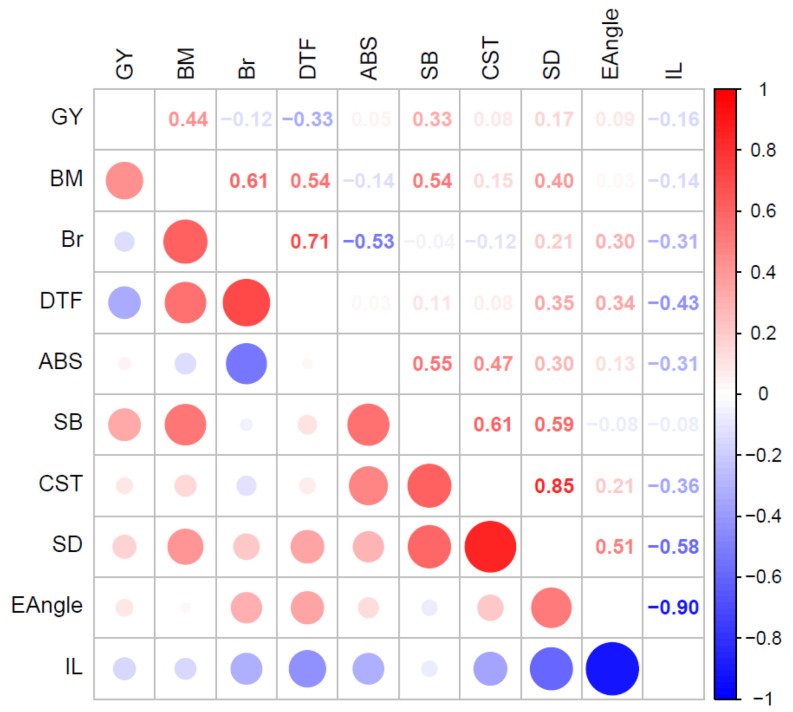
Additive genetic correlations calculated from the additive variance and covariance components from the optimized multivariate linear mixed model of 10 traits: single plant grain yield (GY), dry biomass (BM), basal branches (Br; square root transformed), days to flower (DTF), ascochyta blight score (ABS), stem buckling (SB), compressed stem thickness (CST), stem diameter (SD), early stem angle (EAngle), and internode length (IL). Actual values of the genetic correlations are shown above the diagonal.

The optimized MLMM allowed for covariance of additive genetic effects and residual effects between traits. Covariances of additive effects were converted to correlations (Figure 3). Correlations of residual effects were also significant between many traits and are presented in Supplemental Figure S1.

Strong positive additive genetic correlations were found among traits BM, Br, and DTF (≥0.54) and among the stem strength traits SB, CST, and SD (≥0.59), whereas the strongest negative correlations occurred between EAngle and IL (−0.90) and between IL and SD (−0.58), that is, shorter internodes were associated more upright growth and with broader stems (Figure 3). Lower ABS (higher resistance to ascochyta blight disease) was associated with higher basal branching (Br) (−0.53). ABS showed moderate positive correlations with SB and CST (≥0.47). GY had a moderate correlation with BM (0.44) (Figure 3).

The positive genetic correlations between ABS and SB, and ABS and CST, indicate that stronger stems were associated with higher disease levels, which hinders selection for both strong stems and ABS resistance. SB was positively correlated with DTF which indicates that stronger stems were associated with later flowering.

### 2.5. Accuracy of Predicted Breeding Values

The average accuracy of PBV across all traits in S_0_ progeny significantly increased from 0.799 in the univariate LMM to 0.841 in the optimized MLMM (Table 3) (*t* = −189.9, *p* < 0.001), which was slightly higher than accuracy of PBV in S_2+_ progeny in univariate LMM (0.835) and demonstrates a significant benefit of MLMM over univariate LMM for S_0_ progeny. Furthermore, the average accuracy of PBV for S_2+_ progeny significantly increased to 0.875 in the optimized MLMM (Table 3).

### 2.6. Prediction of Genetic Gain in the Next Cycle with OCS

PBVs for each trait from the optimized MLMM were weighted for desired genetic gains and summed to generate a selection index for each individual. Trait weightings were chosen to increase predicted genetic gains in GY, BM, SB, CST, SD, and EAngle, to reduce DTF and ABS, and to maintain Br and IL (Table 4). The index, pedigree information and list of genotypes available for crossing were submitted to MateSel for OCS with conservative parameters for improving genetic gain in Cycle 4 while minimising the loss in genetic diversity in the population (Table 4). Fine-tuning of the trait weightings occurred in MateSel for ABS and CST to decrease ABS and increase CST. The run achieved predicted genetic gain in GY, BM, and stem strength traits of 0.9 to 9.4% (Table 4) while reducing DTF by 2.5% and ABS by 3.8%. Despite a weighting to maintain IL, the predicted PBV of IL decreased by 10.5% in the next cycle (Table 4). The achieved parental coancestry in Cycle 4 was very low at 0.12.

## 3. Discussion

Multivariate linear mixed models improved the accuracy of predicted breeding values in non-inbred progeny of an experimental field pea population for several traits with low to moderate heritability compared to univariate models. This research shows the value of exploiting the information in correlated traits to improve the accuracy of PBV in non-inbred progeny. Accurate selection in early generations of field pea breeding can accelerate annual genetic gain for these traits.

A bivariate LMM of ABS and DTF in Cowling et al. [18] showed a strong negative correlation between the additive genetic effects for ABS and DTF in Cycle 2 (Supplemental Table S1B), which confirmed a previous report that late flowering field peas tended to have lower ABS (i.e., were more resistant to Ascochyta blight) than early types [9]. The bivariate analysis improved the $h^{2}$ values and accuracy of PBV for ABS over the univariate analysis presented in Cowling et al. [18], and therefore it was used to generate an optimised crossing design to being Cycle 3.

In 2019, the Cycle 3 progeny were evaluated as spaced single S_0_ and S_2+_ plants sown into a weed-suppression mat. This is clearly different from the conditions in which field pea crops are normally grown, but this design permitted an unbiased assessment of trait values on individual progeny plants. The sowing of spaced single plants in a weed mat may affect the growth of the root system and stem due to higher soil temperatures. However, the monthly average temperature of the region ranged from 14 to 20 °C during the winter-spring months in which the trial was grown, and therefore it is unlikely that the weed mat resulted in detrimental effects on root systems due to high soil temperatures. Similarly, sowing of blueberries into a weed mat or sawdust mulch had no negative impact on growth and yield in most years [46]. Wide spacing of single plants was useful to record EAngle and to achieve an unbiased estimate of all traits on individual progeny plants. Similar designs were used in previous studies where high levels of ascochyta blight disease and low to moderate heritability for ABS resistance were recorded [9,18].

Previously, improvements in lodging resistance in field peas resulted from the breeding of dwarf semi-leafless varieties [7,15,22] which have better standability due to tendrils which hold the crop together [23]. Semi-leafless pea plants have weak stems and there has been little or no genetic progress in improving their stem strength. We measured several traits related to stem strength (SB, CST, SD, EAngle, and IL), all of which contribute to stem structure and standability of individual plants, with the goal of making significant genetic progress for stronger stems.

In previous studies, CST and SD were shown to be correlated with physical measures of stem strength such as flexion and force at breaking point [47] and both traits responded to selection [9]. Smitchger and Weeden [20] also found a genetic relationship between lodging resistance and CST and SD, and Smitchger et al. [48] suggested that SD should be the major focus of breeding to increase lodging tolerance. We improved the reliability of CST measurement method over previous methods [20,47] by standardizing the displacement in the stem at a fixed load of 10 N (Figure 2a).

We included a new measurement of stem strength, stem buckling (SB), where the base of the stem was fixed and the top was pressed downwards by the flat end of the force gauge, following the methods in Niklas [49]. SB has been proposed as a good stem strength predictor in plants [50], however, Smitchger et al. [48] considered that pea stems normally do not fail by stem buckling since plants do not usually grow in a vertical position, and attributed an increased lodging tendency to stem angles closer to the horizontal. Hence, we also measured EAngle on individual plants. We found that shorter IL was strongly correlated with higher EAngle (more upright growth), but extremely short internodes are not desirable as this results in short plants and poor harvestability [48]. Our results suggest that it should be possible to breed a more compact pea crop with slightly shorter internodes, with stronger stems and better lodging resistance as predicted by higher EAngle, SB, CST, and SD.

Another component of theoretical column buckling strength is the second moment of area [49]. Future work could include this measurement as an additional indicator of stem strength. It will likely be correlated with SB and indicate the expected buckling type (i.e., Euler or Brazier), which is associated with the wall thickness ratio [51]. However, CST is expected to be a good proxy of second moment of area due to its relationship with stem wall thickness.

In this population, SB, SD, and CST had low narrow-sense heritability (Table 2), but there were moderate to high additive genetic correlations between these traits in the optimized MLMM (Figure 3). Hence, the average accuracy of PBVs in S_0_ progeny from 0.799 to 0.841 across all traits in the optimized MLMM over the corresponding univariate LMM (Table 3). To put this into perspective, the accuracy of PBVs for S_0_ progeny in multivariate models was higher than the accuracy of PBVs in selfed (S_2+_) progeny in univariate models. We conclude that selfing to S_2_ or higher within breeding cycles is not essential to achieve significant genetic gain for these low heritability traits. We achieved annual cycles of S_0_ recurrent selection with high accuracy of PBV based on an optimized MLMM of several correlated low to medium heritability traits. Recurrent selection on S_0_ progeny avoids the cost of selfing and longer breeding cycles when selection occurs on inbred lines. This supports the suggestion of Cobb et al. [1] that shorter cycles and selection on non-inbred progeny should be explored as an option for improving genetic gain in crop breeding.

The optimised MLMM benefited from correlations among additive effects and residuals across traits which improved the data structure in the model and increased the accuracy of PBV, as originally shown by Thompson and Meyer [34]. Similar benefits were obtained with MLMM in Eucalyptus tree breeding [35], where self-pollination does not occur (or is very rare) and all progeny were S_0_ or clones thereof. Benefits of MLMM were also shown in genomic prediction in inbred wheat lines [52].

Our study is one of the first applications of the single step MLMM with pedigree information in self-pollinating crops, and our results support the use of MLMM for several low to medium heritability traits measured on non-inbred progeny to help achieve rapid genetic gain. In this study, we achieved a predicted genetic gain in the next cycle of −3.8% in ABS, +5.0% in CST, +5.1% in SD, and +7.8% in EAngle, while controlling DTF and IL and achieving low parental coancestry of 0.12 (Table 4). Rapid and sustained genetic gain for several traits was predicted in stochastic models of S_0,1_ family selection [53,54], and validated in the field over several cycles of recurrent selection in spring canola for grain yield and several other low to moderate heritability traits [55].

S_0_ recurrent selection [3] may be augmented by including self progeny of parent plants at S_2_ or higher selfing generations (S_2+_). Augmenting S_0_ recurrent selection with S_2+_ selfs of parent plants improved connectivity of genetic relationships between and within cycles, and improved the accuracy of PBV [55]. It is also of practical and commercial value to include S_2+_ selfs of parent plants in augmented S_0_ recurrent selection, as this provides inbred lines ready for commercial evaluation after two cycles. This simplifies the proposed two-part strategy (population improvement and product development) for breeding self-pollinating crops [56] by combining the two parts into a single breeding program.

Our study was based on measurements made in the field, but the accuracy of PBV in non-inbred progeny could also be increased by including additional traits from high-throughput phenotyping in controlled environments to improve the accuracy of PBV for all traits [57].

Genomic relationship information is expected to improve accuracy of PBV especially when combined with pedigree information [52,56,58,59]. It will be relatively simple to combine genomic analysis (GBLUP) and pedigree analysis (ABLUP) in single-step or hybrid BLUP (HBLUP) analysis [60,61,62,63]. GBLUP was used for selection of non-inbred progeny of wheat by Bonnett et al. [64], and we expect that accuracy of PBV on non-inbred progeny will be improved further when pedigree and genomic information are combined in HBLUP.

The inclusion of multiple correlated traits increases the complexity and number of calculations required to converge the MLMM. In this study, we ran our models outside the R environment to overcome the CPU limitations that appeared in the 10-trait model with ASReml-R. Attempting to fit a model with more traits will likely result in convergence errors, so it is worth exploring different approaches of multivariate analysis for large datasets. One alternative would be to divide the analysis into two stages, where estimates of the fixed effects of genotypes (BLUES) are obtained in univariate analyses and are later analyzed in a multivariate BLUP analysis e.g., [65]. In animal breeding studies, correlations between traits in large datasets are sometimes estimated through pair-wise bivariate analyses of all trait combinations e.g., [66]. In theory, if correlations are known beforehand, it would be possible to adjust the multivariate model so that it includes all traits but assumes a value of zero for low and non-significant covariances between traits. In this way, fewer parameters need to be estimated in the model, which should result in easier computation and potentially could achieve convergence in a challenging dataset.

Animal breeders increased the rate of genetic gain by exploiting BLUP models which improved the accuracy of PBV in highly heterozygous individuals [27], but BLUP-based breeding also increased the rate of population inbreeding over cycles. As a result, OCS was implemented in animal breeding to maximize genetic gain whilst controlling the rate of inbreeding [67]. Recently, crop breeders have adopted OCS or similar optimized selection frameworks that retain genetic variability of the population under selection to improve the long-term genetic response [1,55,58,65,68,69,70,71]. OCS was valuable in this study to predict the outcome of MLMM on genetic response for several traits in the next generation based on an optimised mating design.

The MLMM that we implemented here is relatively simple to adopt and involves little additional cost to the breeding program. Pedigree information can be recorded and recalled in an appropriate data base at low cost. Genomic data can be added in the form of a genomic relationship matrix if and when available. The challenge remains to implement MLMM in small plot trials grown at many sites per cycle across multiple cycles of selection, with measurements of GY, lodging, and other commercial traits. So far, this has only been achieved with univariate analyses, one trait at a time, across all sites and cycles [55].

## 4. Materials and Methods

### 4.1. Crossing to Begin Cycle 3

Progeny evaluated in the previous cycle (Cycle 2) of this field pea experimental population [18] were selfed, harvested and stored for later use as parent plants to generate Cycle 3 progeny for evaluation in this study. Cycle 2 data for ascochyta blight score (ABS) [18] and days from sowing to first flower (DTF) (Supplemental Table S1A) were re-analyzed in a bivariate model with pedigree information to generate predicted breeding values (PBVs) for each trait (Supplemental Table S1B). A selection index was constructed with economic weights on PBVs which aimed to reduce ABS while maintaining DTF (Supplemental Table S1C).

Optimal contribution selection (OCS) based on this selection index was implemented in software MateSel [45] (Supplemental Table S1C), which is based on an evolutionary algorithm with constraints easily invoked to ensure practical relevance and precise control of selection response and other outcomes. MateSel dictates which individuals to select and the actual crossing allocations and/or selfings to be made in a crossing design that balances genetic gain for the selection index under the constraint of a maximum permissible target inbreeding rate [44,45,67]. Retained S_1_ seed harvested from S_0_ individuals was used for crossing and selfing of each parent plant, and each crossing and selfing was recorded explicitly in the pedigree at the individual plant level [18].

The resulting mating list generated by MateSel comprised 150 crosses and was based on 60 Cycle 2 parental genotypes (Supplemental Table S1D). Crosses for Cycle 3 were carried out in a glasshouse at The University of Western Australia (UWA) Field Station, Shenton Park, Western Australia, during the summer and autumn months (December 2018 to April 2019). Cycle 3 cross progeny seed (S_0_) along with selfs of the parent plants at S_2_ or higher selfing generations (S_2+_) were harvested and prepared for the Cycle 3 field trial, which forms the basis of this study.

### 4.2. Field Trial and Trait Assessment

In early June 2019, Cycle 3 progeny were grown in a field trial at UWA Field Station. Single plants were sown 90 cm apart in a square grid design 20 rows wide by 80 ranges (columns) deep, based on a spatially-optimized partially replicated design using the R package DiGGer [72]. The population included 668 S_0_ genotypes (from crosses), 612 S_2+_ genotypes (selfs of parents used in crosses) and 320 control plants (replicates of 15 control varieties), resulting in a trial with 1600 single plants (Figure 1a). The field was covered with black plastic weed mat to assist with weed control. One week after germination, infected pea straw with ascochyta blight disease from the previous cycle was spread across the trial to promote even disease infection.

Plants were scored for several traits during the growing season from June to November 2019. Ascochyta blight score (ABS) was measured as the number of nodes on the main stem from the base of the plant with stipules or leaves showing ascochyta blight disease symptoms at first flower, with lower values indicating higher resistance. Other recorded traits included days to first flower (DTF) and number of basal branches (Br). Stem angle above horizontal (EAngle) was assessed before the flowering stage (Figure 1b). After harvest, plants were collected, dried, and weighed for above-ground biomass (BM) and single plant grain yield (GY).

To assess stem strength, the first 15 cm of each main stem was detached at harvest and measured for mean internode length of the first four nodes (IL) and stem diameter (SD) at the center of the third stem internode. Compressed stem thickness (CST) was measured using a digital force gauge (Starr FGD-100, Starr Instruments, Melbourne, VIC, Australia) to apply a force of 10 N at the center of the third stem internode, recording CST as SD less displacement (mm) at 10 N (Figure 2a). Lastly, the top 10 cm of each stem piece was used to assess the stem buckling critical load (SB) in a setup where both ends of the stem were essentially fixed by restricting the base of the stem to the bottom of the digital force gauge with a clamp and pressing the top with the flat attachment of the device (Figure 2b). SB was recorded as the peak force (N) required to produce the structure failure when the force gauge was actuated downwards [49].

### 4.3. Statistical Methods

#### 4.3.1. Univariate Linear Mixed Model

First, all traits were analyzed in a univariate linear mixed model (LMM) where the baseline model accounts for the randomization and is further extended to account for spatial variability in the field trial. Design blocks and genotypes were treated as random effects, and the residual model assumed independent and identically distributed error effects. To account for spatial variability within the trial, in particular the local stationary trend, a first-order separable autoregressive correlation process (AR1 × AR1) for ranges and rows was included, if significant, in the model, following Gilmour et al. [73]. In the model used here, we did not fit the AR1 × AR1 structure in the residual term but as a random effect to maintain a comparable model structure to the multivariate models, where it is not possible to fit the AR1 × AR1 structure in the residual term. In addition, as concluded by De Faveri et al. [74], the separable AR1 × AR1 structure in the residual term may not be adequate for multivariate models, and as a random term this can be dropped if it is found to be not significant. Additional linear fixed and random range and/or row effects were considered and included in each model if the term was significant and did not overfit the data.

The initial LMM was defined as:

(1) $$y=X\beta +Z_{g}u_{g}+Z_{o}u_{o}+e$$

where y is the vector of individual plot observations for a given trait, β is the vector of fixed effects with associated design matrix X, $u_{g}$ is the vector of random genotype effects with associated design matrix $Z_{g}$, $u_{o}$ is the vector of significant random effects other than genotype (AR1 × AR1 structure, range and/or row), with associated design matrix $Z_{o}$, and e corresponds to the vector of plot residual effects. Vectors$\;u_{g}$, $u_{o}$**,** and e, representing random effects, are assumed to follow a Gaussian distribution with zero-mean vector and pairwise independence, so that $u_{g}\sim N\left(0,I⨂G_{g}\right)$, $u_{o}\sim N\left(0,I⨂G_{o}\right)$, and e~N(0,I⨂R), where I is an identity matrix and$\;G_{g}$, $G_{o}$ and R are diagonal type variance-covariance matrices.

A complete pedigree file of the population back to founders was based on the pedigree in Cowling et al. [18] and updated with new crosses and progeny in Cycle 3 (Supplemental Table S2A). A pedigree relationship matrix (A-matrix) was included in the analysis to estimate additive genetic effects (PBV) in each model as previously described [18]. In addition, it was possible to fit a variance component for the non-additive or residual genetic effects. This non-additive term was included only if it was significant and if inclusion increased the log-likelihood of the model. Thus, the LMM with pedigree information was defined as follows:

(2) $$y=X\beta +Z_{g}u_{ga}+Z_{g}u_{gn}+Z_{o}u_{o}+e\;$$

where the model is equivalent to Equation (1), following the same assumptions, but the component $u_{g}$ is partitioned into $u_{ga}$ as the vector of random additive genetic effects and $u_{gn}$ as the vector of random non-additive genetic effects with $u_{ga}\sim N\left(0,A⨂G_{g}\right)$ and $u_{gn}\sim N\left(0,I⨂G_{g}\right)$, where A corresponds to the A-matrix of pedigree relationship information.

Each successive model was evaluated based on changes in log-likelihood, Akaike information criterion (AIC), and Bayesian information criterion (BIC). Comparisons between models were made through a log-likelihood ratio test. The significance of fixed effects was assessed by a Wald test. Random terms were retained when they significantly improved the log-likelihood of the model. Narrow-sense heritability ($h^{2}$) for each trait was calculated from the estimated variance components from the respective LMM with pedigree information, where $h^{2}$ equals the additive variance component divided by the sum of the additive, non-additive and residual variance components.

#### 4.3.2. Multivariate Linear Mixed Model

The second approach was to fit a multivariate linear mixed model (MLMM) across traits, constructed by specifying a matrix of combined traits as the response [75]. Different variance structures can be fitted for each random and residual term specified inside the MLMM, thus, it is possible to obtain independent variances for the different effects of each trait and covariances between trait effects. This allows for a greater level of fine-tuning when modelling because the covariance between trait effects can be allowed or omitted independently for the different terms in the model.

In MLMM, the process began by fitting a baseline multi-trait model, using diagonal variance structures (DIAG) for the genotype and residual terms, which assumes that traits are independent (covariance between traits is zero) and have separate variances. This is comparable to fitting parallel univariate models. Second, the MLMM was improved by assuming an unstructured covariance model (US), instead of a DIAG variance structure, for the genotype and residual terms, permitting the estimation of covariances for these terms between traits [75]. Finally, pedigree relationship information in the form of an A-matrix was added to the MLMM, enabling the estimation of PBV, as in the univariate step.

For each trait present in the model, the AR1 × AR1 structure and other range or row random effects for each trait were fitted separately and retained in the final model only if significant. This means that it was possible to test and fit these terms in a trait-by-trait case. For example, the AR1 × AR1 structure was modelled only for ABS, DTF had a random row effect and GY had both a random row and range effect. Likewise, the non-additive genetic term was included in the MLMM or omitted depending on its significance for each trait and was included as either a US or DIAG structure, based on the significance of the covariance term (meaning that it was possible to create a model with covariance between additive genetic effects, but non-additive genetic effects were assumed independent). The final multivariate model was defined as:

(3) $$Y=XB+Z_{g}U_{ga}+Z_{g}U_{gn}+Z_{o}U_{o}+E$$

where terms are as per the univariate model in Equation (2) but expanded for additional traits, so that for *N* traits

$$Y=\left[\begin{array}{c} Y_{1}\\ \vdots \\ Y_{N} \end{array}\right]$$

and Y is the combined vector of line observations for N traits included in the model, B is the matrix of combined vectors of significant fixed effects for each trait identified in the univariate analyses with associated design matrix X, $U_{ga}$ is the combined vector of random additive genetic effects with associated design matrix $Z_{g}$, with $U_{ga}\sim N\left(0,A⨂T_{a}\right)$, $U_{gn}$ is the combined vector of random non-additive genetic effects with associated design matrix $Z_{g}$, with $U_{gn}\sim N\left(0,I⨂T_{n}\right)$, $U_{o}$ is the combined vector of significant random effects other than genotype for each trait, with associated design matrix $Z_{o}$, and E corresponds to the matrix of residual effects with E~N(0,I⨂R). A is the A-matrix of pedigree relationship information, I is an identity matrix and $T_{a}$, $T_{n}$**,** and R are the trait variance-covariance matrices of additive, non-additive, and residual effects, respectively.

$T_{a}$ and R were modeled with a US variance structure resulting in the additive genetic and residual covariance of trait effects such as

$$T_{a}=\left[\begin{array}{ccc} \sigma_{Ga11}^{2} & \cdots & \sigma_{Ga1N}\\ \vdots & \ddots & \vdots \\ \sigma_{Ga1N} & \cdots & \sigma_{GaNN}^{2} \end{array}\right]\;\mathrm{and}\;R=\left[\begin{array}{ccc} \sigma_{R11}^{2} & \cdots & \sigma_{R1N}\\ \vdots & \ddots & \vdots \\ \sigma_{R1N} & \cdots & \sigma_{RNN}^{2} \end{array}\right]$$

However, $T_{n}$ assumed a US variance structure only if the covariance of non-additive genetic effects between traits was significant, otherwise a DIAG variance structure was specified and non-additive genetic effects were assumed independent, resulting in either

$$T_{n}=\left[\begin{array}{ccc} \sigma_{Gn11}^{2} & \cdots & \sigma_{Gn1N}\\ \vdots & \ddots & \vdots \\ \sigma_{Gn1N} & \cdots & \sigma_{GnNN}^{2} \end{array}\right]\;\mathrm{or}\;T_{n}=\left[\begin{array}{ccc} \sigma_{Gn11}^{2} & \cdots & 0\\ \vdots & \ddots & \vdots \\ 0 & \cdots & \sigma_{GnNN}^{2} \end{array}\right]$$

for US variance structure and DIAG variance structure, respectively.

The covariances of trait effects estimated in the model were transformed to correlations using the variance value for each trait and their covariance:

(4) $$\rho_{XY}=\frac{\sigma_{XY}}{\sqrt{\sigma_{X}^{2}*\sigma_{Y}^{2}}}\;$$

where $\rho_{XY}$ is the correlation coefficient between the effect of traits X and Y, $\sigma_{XY}$ is the covariance between the effects of traits X and Y, and $\sigma_{X}^{2}$ and $\sigma_{Y}^{2}$, are the variances of traits X and Y, respectively.

As in the univariate process, all fitted MLMM were evaluated in terms of log-likelihood, AIC, and BIC, using a log-likelihood ratio test to compare models. The significance of fixed effect terms was assessed by a Wald test. Random terms were retained if they produced a significant improvement in the log-likelihood of the model. $h^{2}$ for each trait was calculated using the estimated variance components of the MLMM as the additive variance component divided by the sum of the additive, non-additive (if present), and residual variance components for each trait.

The models presented in this study were fitted using the R package ASReml-R version 4 (VSN International Ltd., Hemel Hempstead, UK) [75]. For the more complex models, the MLMM was optimized in the stand-alone version of ASReml version 4.2 (VSN International Ltd., Hemel Hempstead, UK) [76] due to its ability to use more CPU cores in the run.

#### 4.3.3. Model Accuracy

The accuracy of PBV obtained from the final models was calculated as the correlation between the true and predicted breeding values based on the approach of Mrode [77]:

(5) $$r_{pi}=\sqrt{1-\frac{s_{i}^{2}}{\left(1+f_{i}\right)\sigma_{A}^{2}}}$$

where the accuracy $r_{pi}$ of prediction for the individual *I*, $s_{i}^{2}$ is the squared standard error that accompanies the breeding value of individual *i* obtained from the model prediction, $f_{i}$ is the inbreeding coefficient calculated based on the pedigree for individual *i* and $\sigma_{A}^{2}$ is the trait additive genetic variance estimated from the model [76].

The average accuracy of PBV of groups of progeny S_0_ and S_2+_ were compared across models.

### 4.4. Prediction of Progeny Performance in Cycle 4

PBVs for each trait were obtained from the optimized MLMM. An optimized selection index for each individual was calculated from the sum of weighted PBVs across traits, where the weights for each trait were based on the tactical desired gains approach [78] and calculated in the program DESIRE [79]. The selection index and the PBVs for each trait for each individual were submitted to software MateSel to generate an optimized mating design with 170 crosses to begin the fourth cycle. The simulation ran with a weight against progeny inbreeding of −0.01 and a conservative target of 50 degrees in the response frontier of the population to promote a moderate increase in index while minimizing achieved population coancestry [45]. The output summary included predicted changes in mean index value, PBVs and achieved parental coancestry in the matings to begin the fourth cycle.

## 5. Conclusions

The accuracy of predicted breeding values for 10 low to medium heritability traits in non-inbred progeny of field pea increased in an optimized multivariate linear mixed model over a univariate model, making it feasible to undertake annual cycles of S_0_ recurrent selection on non-inbred progeny. Significant genetic gains in several low heritability traits were predicted in the next cycle with low rates of population inbreeding based on a mating design generated with optimal contribution selection. The results show that the information contained in correlated traits can be exploited to increase accuracy of selection in early generations of field pea breeding.

## Figures and Tables

**Table 1:** Additive genetic, non-additive genetic, and residual variance components from univariate linear mixed model analyses (LMM) for 10 traits scored in Cycle 3 of the experimental field pea breeding population.

Trait Abbreviation	Univariate LMM Variance Components ± SE			*h*^2^ ± SE
	Additive	Non-Additive	Residual	
GY	22.79 ± 8.15	103.77 ± 14.64	79.60 ± 10.98	0.11 ± 0.04
BM	77.79 ± 26.55	232.02 ± 46.36	279.74 ± 37.21	0.13 ± 0.04
Br	0.08 ± 0.01	ns	0.14 ± 0.01	0.36 ± 0.05
DTF	24.14 ± 3.89	11.05 ± 1.34	8.46 ± 0.72	0.55 ± 0.05
ABS	0.62 ± 0.16	ns	5.81 ± 0.24	0.10 ± 0.02
SB	2.01 ± 0.71	ns	31.39 ± 1.46	0.06 ± 0.02
CST	0.04 ± 0.01	0.07 ± 0.03	0.19 ± 0.03	0.13 ± 0.04
SD	0.04 ± 0.01	ns	0.23 ± 0.02	0.15 ± 0.04
EAngle	98.76 ± 21.05	ns	327.06 ± 13.44	0.23 ± 0.04
IL	0.48 ± 0.08	0.11 ± 0.04	0.31 ± 0.03	0.53 ± 0.05

Abbreviations: GY, single plant grain yield; BM, dry whole plant biomass at maturity; Br, number of basal branches (square root transformed); DTF, days to flower; ABS, ascochyta blight score; SB, stem buckling; CST, compressed stem thickness; SD, stem diameter; EAngle, early stem angle from horizontal; IL, internode length; LMM, linear mixed model; *h*^2^, narrow-sense heritability; ns, not significant; SE, standard error.

**Table 2:** Additive genetic, non-additive genetic, and residual variance components from the optimized multivariate linear mixed model (MLMM) analysis for 10 traits scored in Cycle 3 of the experimental field pea breeding population.

Trait Abbreviation	MLMM Variance Components ± SE			*h*^2^ ± SE
	Additive	Non-Additive	Residual	
GY	35.90 ± 10.23	18.78 ± 4.62	245.70 ± 12.16	0.12 ± 0.03
BM	99.43 ± 29.24	ns	797.45 ± 54.54	0.11 ± 0.03
Br	0.09 ± 0.02	ns	0.17 ± 0.01	0.35 ± 0.04
DTF	32.22 ± 4.50	7.51 ± 1.20	10.00 ± 0.82	0.65 ± 0.04
ABS	0.61 ± 0.14	ns	5.90 ± 0.24	0.09 ± 0.02
SB	1.81 ± 0.61	ns	32.96 ± 1.46	0.05 ± 0.02
CST	0.04 ± 0.01	ns	0.28 ± 0.01	0.12 ± 0.03
SD	0.04 ± 0.01	ns	0.28 ± 0.01	0.14 ± 0.03
EAngle	125.04 ± 21.52	ns	146.15 ± 62.46	0.46 ± 0.12
IL	0.62 ± 0.09	ns	0.39 ± 0.02	0.61 ± 0.04

Abbreviations: GY, single plant grain yield; BM, dry whole plant biomass at maturity; Br, number of basal branches (square root transformed); DTF, days to flower; ABS, ascochyta blight score; SB, stem buckling; CST, compressed stem thickness; SD, stem diameter; EAngle, early stem angle from horizontal; IL, internode length; MLMM, multivariate linear mixed model; *h*^2^, narrow-sense heritability; ns, not significant; SE, standard error.

**Table 3:** Comparison of mean accuracy of predicted breeding values (PBV) ± standard deviation between univariate and optimized multivariate linear mixed models for cross progeny (S_0_) and selfed lines (S_2+_).

Trait	S_0_		S_2+_	
	Univariate	Multivariate	Univariate	Multivariate
GY	0.75 ± 0.06	0.80 ± 0.04	0.79 ± 0.08	0.84 ± 0.06
BM	0.77 ± 0.05	0.81 ± 0.04	0.80 ± 0.08	0.84 ± 0.05
Br	0.86 ± 0.03	0.88 ± 0.02	0.89 ± 0.05	0.91 ± 0.04
DTF	0.88 ± 0.02	0.90 ± 0.02	0.92 ± 0.04	0.94 ± 0.03
ABS	0.77 ± 0.05	0.83 ± 0.04	0.81 ± 0.07	0.86 ± 0.05
SB	0.71 ± 0.06	0.77 ± 0.05	0.74 ± 0.09	0.81 ± 0.06
CST	0.76 ± 0.05	0.81 ± 0.04	0.80 ± 0.07	0.84 ± 0.05
SD	0.78 ± 0.05	0.83 ± 0.04	0.82 ± 0.07	0.86 ± 0.04
EAngle	0.84 ± 0.04	0.88 ± 0.02	0.88 ± 0.04	0.92 ± 0.02
IL	0.87 ± 0.03	0.90 ± 0.02	0.91 ± 0.03	0.93 ± 0.02
Average	0.799	0.841	0.835	0.875

Abbreviations in table: GY, single plant grain yield; BM, dry whole plant biomass at maturity; Br, number of basal branches (square root transformed); DTF, days to flower; ABS, ascochyta blight score; SB, stem buckling; CST, compressed stem thickness; SD, stem diameter; EAngle, early stem angle from horizontal; IL, internode length.

**Table 4:** Predictions of genetic gains in index and PBVs in the next cycle of the experimental field pea population, from optimal contribution selection using MateSel [45]. The population phenotypic means for each trait are shown in Supplemental Table S2B.

Parameter	Units	Selection Goal	Selection Index Weights	Mean PBV in Candidates	Prediction of Mean PBV in Next Cycle	Change in Mean PBV in Next Cycle	Change in Mean PBV in Next Cycle as % of Phenotypic Mean
Index		Increase		2.30	4.55	2.25	
GY	g	Increase	−0.028	2.87	5.46	2.60	+9.4
BM	g	Increase	0.150	2.21	2.76	0.55	+1.0
Br	number	Decrease	−6.000	0.12	0.14	0.02	+0.9
DTF	days	Decrease	−0.200	−2.26	−4.04	−1.79	−2.5
ABS	number	Decrease	−2.900	−0.51	−0.71	−0.20	−3.8
SB	N	Increase	1.200	0.13	0.26	0.13	+1.4
CST	mm	Increase	1.700	0.02	0.13	0.11	+5.0
SD	mm	Increase	2.000	0.08	0.22	0.14	+5.1
EAngle	degrees	Increase	0.150	3.24	8.43	5.19	+7.8
IL	cm	Maintain	−0.030	−0.05	−0.33	−0.28	−10.5

Abbreviations: PBV, predicted breeding value; GY, single plant grain yield; BM, dry whole plant biomass at maturity; Br, number of basal branches (square root transformed); DTF, days to flower; ABS, ascochyta blight score; SB, stem buckling; CST, compressed stem thickness; SD, stem diameter; EAngle, early stem angle from horizontal; IL, internode length.

## Data Availability

The authors affirm that all data necessary for confirming the conclusions of the article are present within the article, figures, tables and supplemental information.

## Supplementary Materials

The following supporting information can be downloaded at: https://www.mdpi.com/article/10.3390/plants12051141/s1, Supplemental Table S1A. List of genotypes and phenotypic records in Cycle 2. Supplemental Table S1B. Cycle 2 bivariate model. Supplemental Table S1C. Cycle 2 MateSel model output. Supplemental Table S1D. Cycle 2 mating list for Cycle 3. Supplemental Table S2A. List of genotypes and phenotypic records in Cycle 3. Supplemental Table S2B. Cycle 3 phenotypic data summary. Supplemental Table S3. Cycle 3 univariate models. Supplemental Table S4A. Cycle 3 base multivariate model. Supplemental Table S4B. Cycle 3 optimized multivariate model. Supplemental Figure S1. Correlation of residuals from Cycle 3 optimized multivariate model.
