# Supplementary material for: Accuracy of Selection in Early Generations of Field Pea Breeding Increases by Exploiting the Information Contained in Correlated Traits
Source: Plants (Basel). 2023 Mar 2;12(5):1141. doi: 10.3390/plants12051141 (PMC10005560; doi:10.3390/plants12051141)
Supplement: Supplementary file 1 [file plants-12-01141-s001.zip › Supplemental Figure S1 - Residual correlations of optimized multivariate model.pdf]

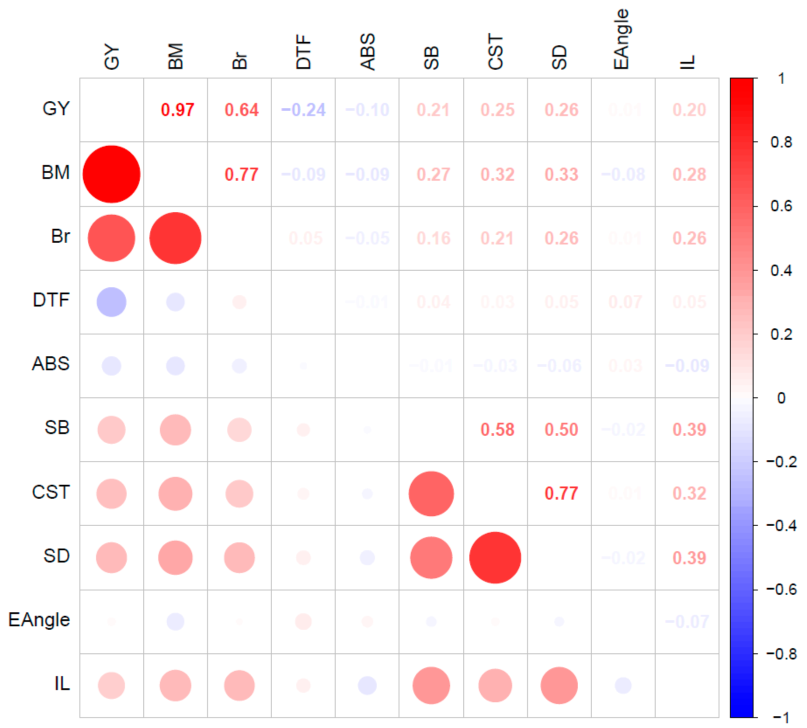


Supplemental Figure S1. Residual correlations from the optimized multivariate linear mixed model of 10 traits: single plant grain yield (GY), dry biomass (BM), basal branches (Br; square root transformed), days to flower (DTF), ascochyta blight score (ABS), stem buckling (SB), compressed stem thickness (CST), stem diameter (SD), early stem angle (EAngle) and internode length (IL). Correlations were calculated using the residual variance and covariance components of the multivariate model output.
